# A Remarkable Case of a Diseased Testicle Successfully Treated

**Published:** 1783

**Authors:** 


II. *A remarkable cafe of a difeafed Tefticle fuccefsfully treated. Communicated to Dr.* Simmons, *F. R. S. by* Robert Hamilton, *M. D. Member of the Medical Society at Edinburgh.*

MUCH has been faid for and againft the Cicuta fince the writings of Störck firft appeared in its favour. The following cafe proves it to be a medicine of confiderable efficacy.

A drummer of the tenth regiment of foot, aged forty-four years (twenty-fix of which he has paffed in the army) with a conftitution impaired by by ſevere ſervice in different climates, and by the frequent uſe of mercury, complained for a long time of ſevere pains in the middle of the large bones, ſuch as the humerus, femur and tibia; and at length entirely loſt the uſe of his left arm, which became much waſted.

To add to his calamities he had a ſwelled teſticle, and that for a greater length of time than he could exactly tell, for it had been better and worſe, he ſaid, at different times, according as he had been circumſtanced; but he ſpoke of it as a complaint of many years ſtanding.

About eighteen months ago he was confined on account of this teſticle at Tynemouth. Saturnine poultices were applied to the ſcrotum, and his complaint grew ſomewhat better; but the regiment being ordered to the ſouthern part of the kingdom he was obliged to march. From that time till laſt December I heard no more of him. He then came into the hoſpital in a dreadful ſtate. His left teſticle, he ſaid, had continued more or leſs ſwelled ſince he left the North; and when we ſaw him in the hoſpital its bulk was ſuch, that it would have weighed perhaps four or five pounds.

It reſembled a piece of a large gut ſtuffed, with a bend downwards. The blood veſſels appeared finely ramified and full on the ſcrotum, except at the upper part near the groin, and in the courſe of the ſpermatic cord. This part was ſomewhat more protuberant than the reſt. I applied Goulard's extract of Saturn for about ten days, but without any good effect. The protuberance increaſed, became pointed, ſoft, and yieldyielding, ſo that it ſeemed as if a ſuppuration had taken place, and yet there was no appearance of redneſs or inflammation on the ſkin. At length however it was become ſo large and the fluctuation ſo evident, though ſtill without any change in the colour of the ſkin, that I determined to hazard a puncture.

The integuments were much thickened, and I was not without apprehenſion of wounding ſome of the larger veſſels of the tunica vaginalis, which I knew would be attended with a great deal of trouble, if not danger; and I could not avail myſelf of the caution given by a reſpectable author in ſuch caſes, of darkening the room and holding a candle on the oppoſite ſide of the ſcrotum in order to ſee and avoid them, ſo condenſed were the coats of the ſcrotum. I made a puncture, however, and ſucceeded well. I drew off near two pounds of a greeniſh ſerum, which reduced the teſticle to about half its former bulk.

It was only now that I learned the true ſtate of the teſticle; I could feel it diſtinctly, yet it adhered to the ſcrotum and vaginal coat, except at one part. In its ſhape it reſembled a turnip, but it was ſomewhat more oblong. It was thin, flat, and as hard as a piece of wood, ſo that not the ſmalleſt doubt of its being ſchirrous remained.

I now put him on a courſe of Hemlock powder, prepared from the leaves of the plant. This medicine, which was firſt recommended to me by my friend Dr. Macqueen, a very ingenious and reſpectable phyſician at Yarmouth, I had already already experienced good effects from in feveral cafes. I began with a fcruple, but foon increafed the dofe to a drachm once a day. He had not taken it above a fortnight when I perceived an evident change in the cafe for the better. The adhefions were beginning to give way, and the hard edges were growing round and foft. The puncture foon healed, and no farther collection of matter took place. He continued the ufe of the remedy till within thefe few days; the tefticle gradually decreafed in fize, and two days ago, when he was difcharged from the hofpital, after having been there about eleven weeks, it was very little larger than the other. I wifhed to have made his cure ftill more complete, and procured leave from the Colonel to keep him in the hofpital fix weeks longer, if he chofe to remain there fo long, but he thought himfelf too well to require any more medicine. He is now going to undertake a long journey on foot, which will probably endanger a return of his complaint. He is obliged to go to Chelfea-hofpital to pafs for a penfion, and from thence to his relations, who live at a great diftance from London.

It is remarkable, that both his urine and ftools were coloured by the medicine, as both were as green as grafs while he took it. His arm is in the fame ftate as before.

I think it right to obferve, that during the whole time he was under the Hemlock courfe, he took a quarter of a grain of corrofive fublimate in folution daily. I mention this circumftance, that you may determine how far it probably concontributed to the effects produced. For my own part I do not think that it had any great ſhare in the cure.

*Yarmouth, March* 14, 1783.

